# Report of Cases

**Published:** 1869-04

**Authors:** J. M. D. Greene

**Affiliations:** Register


### Report of Cases.

Under treatment in the Elmira Eye & Ear Institute, for the Quarter ending April 1st, 1869:

| | |
|---|---|
| Under treatment for Granular Lid | 8 |
| " " " Scrofulous Ophthalmia, | 4 |
| " " " Gonorrhœal, | 1 |
| " " " Glaucoma, | 2 |
| " " " Catarrhal Ophthalmia | 4 |
| " " " Simple Conjunctivitis, | 6 |
| " " " Suppurative Keratitis, | 5 |
| " " " Meibomitis | 2 |
| " " " Astigmatism, | 2 |
| " " " Opacity of Cornea, | 3 |
| " " " Incised wound of Cornea, | 8 |
| " " " Otorrhœa, | 4 |
| " " " Deafness, | 6 |
| " " " Catarrh and Disease of Throat | 6 |
| " " " Eczema, | 6 |
| Operated upon for Cataract, | 2 |
| " " " Iridectomy, | 4 |
| " " " Dislodged Lens, | 1 |
| " " " Inversion of Eyelids, | 2 |
| " " " Pterygium, | 4 |
| " " " Strabismus, (Cross Eye,) | 5 |
| " " " Obstruction Lachrymal Duct, | 4 |
| " " " Club Foot, | 2 |
| " " " Cleft Palate, | 1 |
| " " " Removal of Tumors, | 6 |
| " " " Hœmorrhoids | 4 |
| " " " Stone in Bladder, | 1 |
| " " " Tongue Tie, | 1 |
| " " " Amputation Seirrhus Breast, | 1 |
| " " " Hernia, | 1 |
| " " " Scirrhus Uterus, | 1 |
| " " " Nasal Polypii, | 2 |
| " " " Epithelioma, lower lip, | 2 |
| " " " Hydatids of Uterus, | 1 |
| " " " Palpebraral Abcess, | 1 |
| " " " Ovarian Tumor, | 2 |
| Insertion of Artificial Eyes, | 2 |
| Minor Surgical Operations, | 6 |
| Total number of Cases, | 122 |

J. M. D. GREENE, Register.

---

LOVE—A complaint of the heart growing out of an inordinate longing after something difficult to obtain. It attacks persons of both sexes, generally between the ages of fifteen and thirty; some have been known to have it at the age of sixty.

*Symptoms*—Absence of mind; giving things wrong names; calling tears nectar and sighs zephyrs; gazing on the moon and stars; loss of appetite; neglect of business; a loathing for all things—save one; and a constant desire to sigh.

*Effects*—A strong head-ache; pulse high; stupidly eloquent eyes; sleeplessness, and all that sort of thing. At times, imagination bright; bowers of roses; winged cupids; and then, again, oceans of despair, racks, tortures, and hair-triggered pistols.

*Cure*—Get married. If that don't cure you, it will at least open your eyes.—*Punch.*

---

NOT RESPONSIBLE.—We do not hold ourselves responsible for the actions of any parties canvassing for the BISTOURY, save those whose names are mentioned as regular agents.

You, of course, can tell whether to trust your neighbors, who may canvass for it.

